# Understanding retention in longitudinal surveys conducted by mobile phone in lower-resource contexts: A case study from Kenya

**DOI:** 10.1371/journal.pone.0354780

**Published:** 2026-08-31

**Authors:** Corrina Moucheraud, Boyu Fan, Guanghao Li, Eric Ochieng, George Ganda, Ginger Golub, Alex Dahlen

**Affiliations:** 1 Department of Public Health Policy and Management, New York University School of Global Public Health, New York, United States of America; 2 Department of Biostatistics, New York University School of Global Public Health, New York, United States of America; 3 Innovations for Poverty Action, Nairobi, Kenya; Public Library of Science, UNITED KINGDOM OF GREAT BRITAIN AND NORTHERN IRELAND

## Abstract

Mobile phone-based surveys are increasingly used for data collection in global contexts – and although the strengths and limitations of this methodology in high-income countries are well-documented, less is known about using this approach (particularly for longitudinal data collection) in low- and middle-income countries, where mobile phone ownership has expanded rapidly. In this paper, we leverage data from a parent study about cervical cancer prevention to understand the features associated with retention and loss to follow-up in repeated random-digit dialed mobile phone-based surveys over time in Kenya. From a cohort of 1405 people surveyed in 2022, 1167 were found again in 2024 (83.1%) and 1106 completed the 2024 survey (78.7%). Survey re-completion was significantly associated with older age (versus 17−32 year olds: OR 1.63 [95% CI 1.21–2.20] for 33−38 year olds, OR 1.65 [95% CI 1.22–2.22] for 39−45 year olds, and OR 2.62 [95% CI 1.84–3.72] for ≥46 year olds), male gender (OR 1.44 [95% CI 1.14–1.83]), and household income. Only age was significantly associated with our secondary outcome of being retained in the sample for follow-up (regardless of survey re-completion). This study’s high follow-up rate was not substantially different across these aforementioned groups, suggesting that mobile phone-based data collection is a promising approach, even for longitudinal surveys, in diverse global settings. We encourage more research about, and using, this methodology so researchers can better understand, and improve, follow-up and its determinants.

## Introduction

There is increasing use of remote data collection methods, like mobile phone-based surveys, in a variety of global contexts [1–4]. The strengths and limitations of the mobile phone survey methodology in high-income countries have been well-documented [3,5–9]. These surveys can improve efficiency and lower costs, as well as potentially reduce certain reporting and measurement errors; but they can also introduce potential sources of bias, including non-representativeness and non-response. There has, however, been less empirical data on this methodological issue from low- and middle-income countries (LMICs) [10–14], where mobile phone penetration has exploded [15] and the potential for mobile phone-based data collection is great. In addition, emergency circumstances (like pandemics or natural disasters) may necessitate remote data collection approaches [9,11,16,17], so it is important to understand the potential for these methodologies in diverse global settings [18,19]. Longitudinal studies – wherein the same respondent provides multiple data points over time – have enormous potential for providing meaningful data particularly for time-varying exposures and outcomes [11,20–23], and may be particularly advantaged by using mobile phone-based surveys as these would reduce costs and complexities related to multiple rounds of in-person data collection. These are especially promising in settings with frequent migration where retention in in-person surveys may therefore be low.

There are numerous potential sources of bias that are unique to mobile phone-based surveys and may result in response error [24,25] and differences in call outcomes [25] – including call screening, reaching respondents at a bad time, respondents changing numbers, or reaching the wrong person. These may be particularly relevant to studies using a longitudinal design as there are many reasons why a study participant might be lost over time (attrition), including changing their number, not answering the phone, or not consenting to continue with the study. Previous longitudinal mobile phone surveys in LMICs have achieved high response rates over time (>90%) [11,21,23,26,27] and have found varying evidence of differential attrition across subgroups. Several studies from LMICs have found that attrition varies by age (although some have found greater attrition among older people [28] while others have found the opposite [29,30]), respondent or household head educational attainment [28–31], household location [18,28,30,32,33], and household size [31,34]; some studies have found differential attrition by household wealth [18,28,30,31] but others have not [29,33], and some have found differences by gender [32] and others have not [27].

In this paper, we seek to describe a study population of Kenyan adults that was selected and enrolled using random-digit dialing of mobile phones; they were first surveyed in 2022 and then followed up with in 2024. We analyze whether there is evidence of differential attrition from the study over this two-year period. Nearly all Kenyans personally own a mobile phone and use it daily [35]; due to this near-universal ownership of mobile phones, Kenya is an optimal environment to understand response and attrition patterns in mobile phone-based surveys.

## Methods

This is an analysis of data collected from two surveys with a sample of Kenyan adults. The parent study was focused on factors associated with vaccination against human papillomavirus [36] so all respondents were parents or guardians of preadolescent and early adolescent girls, i.e., currently or recently age-eligible for the vaccine. These respondents were enrolled and surveyed in 2022, and re-contacted again in 2024, forming a longitudinal panel of participants across the two waves. Here we analyze patterns in re-response rates to explore factors associated with follow-up in the cohort over this period.

### Study setting

Kenya is a country of approximately 57 million people in eastern Africa. Per the most recent Afrobarometer data, approximately 95% of Kenyan respondents personally own a mobile phone and 88% of them use it daily; and there are only small subgroup differences: mobile phone ownership was 97.7% among urban respondents and 93.2% among rural respondents, and 95.6% among males and 93.7% among females [35]. This very high coverage of mobile phones made it an appropriate setting for this study about phone survey response rates.

### Data collection

Between July and October 2022, we conducted a phone survey in Kenya. We obtained a list of 25,000 random computer-generated mobile phone numbers, from which we pulled numbers to call. Specifically, we identified valid mobile network prefixes and for each prefix, the remaining digits were populated using a pseudo-random number generator. To avoid systematic bias, numbers were generated independently without replacement, and the final sample was drawn randomly from the pool of successfully reached and eligible respondents. All procedures were pre-specified and implemented programmatically to ensure reproducibility.

When a call attempt was successful, a trained research assistant asked the person if they were interested in learning about the study; those who expressed interest were then assessed for eligibility – i.e., identified as a parent or guardian for a girl aged 10–16 years. If the respondent was interested and eligible, they gave oral informed consent (including to be contacted for future studies) before beginning the survey. The surveys asked about knowledge, attitudes and experiences with HPV vaccination and cervical cancer screening for eligible girls and women in the household, as well as demographic and household information. On average the surveys conducted in 2022 were 27.6 minutes in duration (SD 9 minutes).

People who completed the 2022 survey were re-contacted between May and July 2024, approximately two years later. The same phone numbers were dialed, and the trained research assistant asked to speak with the original respondent. If identity was confirmed, the assistant then assessed whether the respondent was willing to participate in the follow-up survey and obtained oral consent before proceeding with the 2024 questionnaire. This survey replicated questions from the 2022 survey (updated to include girls newly aged-in to HPV vaccination eligibility when relevant) plus a new module about sharing health information within social networks; on average this survey lasted 33.8 minutes (SD 23 minutes).

### Key variables

We defined retention in two ways. Our primary retention endpoint was “completed follow-up,” defined as participants who completed the full follow-up process: (1) they consented to the follow-up (2024) survey when taking the original (2022) survey; (2) we were able to reach them on the phone and confirm they were the same individual; and (3) they completed the follow-up survey. A secondary retention endpoint was “retained respondents,” defined more broadly as participants who completed steps (1) and (2), but not necessarily (3), meaning that we were able to reach by phone in 2024 and confirm as the same individual, regardless of whether they completed the follow-up survey. By definition, those who completed follow-up are a subset of retained respondents.

We considered the following demographic features as potential factors associated with follow-up or retention: age, gender, area of residence, education, employment status, household income sufficiency over the past 12 months (self-reported insufficient to meet expenses, just enough to meet expenses, or more than enough to meet expenses), marital status, and religion.

### Data analysis

We paired respondents’ data from the 2022 and 2024 surveys using a unique identifier assigned to their phone number (a unique identifier was used in order to maintain a de-identified dataset per data protection and privacy restrictions).

To evaluate associations between participant demographics and retention, we fit multivariable logistic regression models separately for our two outcomes: the primary outcome of repeat respondents (participants reached, consented, and completed the follow-up survey) and the secondary outcome of retained respondents (participants successfully reached and identity-confirmed, regardless of survey completion). Missing demographic data were rare (<10% across all predictors) and were addressed using multiple imputation by chained equations (MICE), with predictive mean matching and 20 replications. We report adjusted odds ratios and pooled 95% confidence intervals and p-values for each association; to account for multiple comparisons, we applied the Benjamini-Hochberg procedure to control the false discovery rate at 10%. All analysis code is publicly available at https://github.com/BoyuFan1/Retention_in_longitudinal_phone_surveys.

### Ethical review

The surveys were reviewed and approved by the University of California Los Angeles Institutional Review Board (#22−000005), and the Kenya Medical Research Institute Scientific and Ethics Review Unit (#SERU4456). As these were telephone-based surveys, informed consent was obtained orally from all participants. At the beginning of each call, enumerators read a standardized consent script in the respondent’s preferred language, which described the purpose of the study, the voluntary nature of participation, procedures involved, potential risks and benefits, confidentiality protections, and the right to decline or withdraw at any time without penalty. Participants were then asked to indicate their willingness to participate, and oral consent was recorded electronically prior to proceeding with the survey. This consent procedure, including audio recording of consent, was explicitly approved by both Institutional Review Boards.

### Inclusivity in global research

Additional information regarding the ethical, cultural, and scientific considerations specific to inclusivity in global research is included in the Supporting Information (S1 File).

## Results

**Fig 1:**
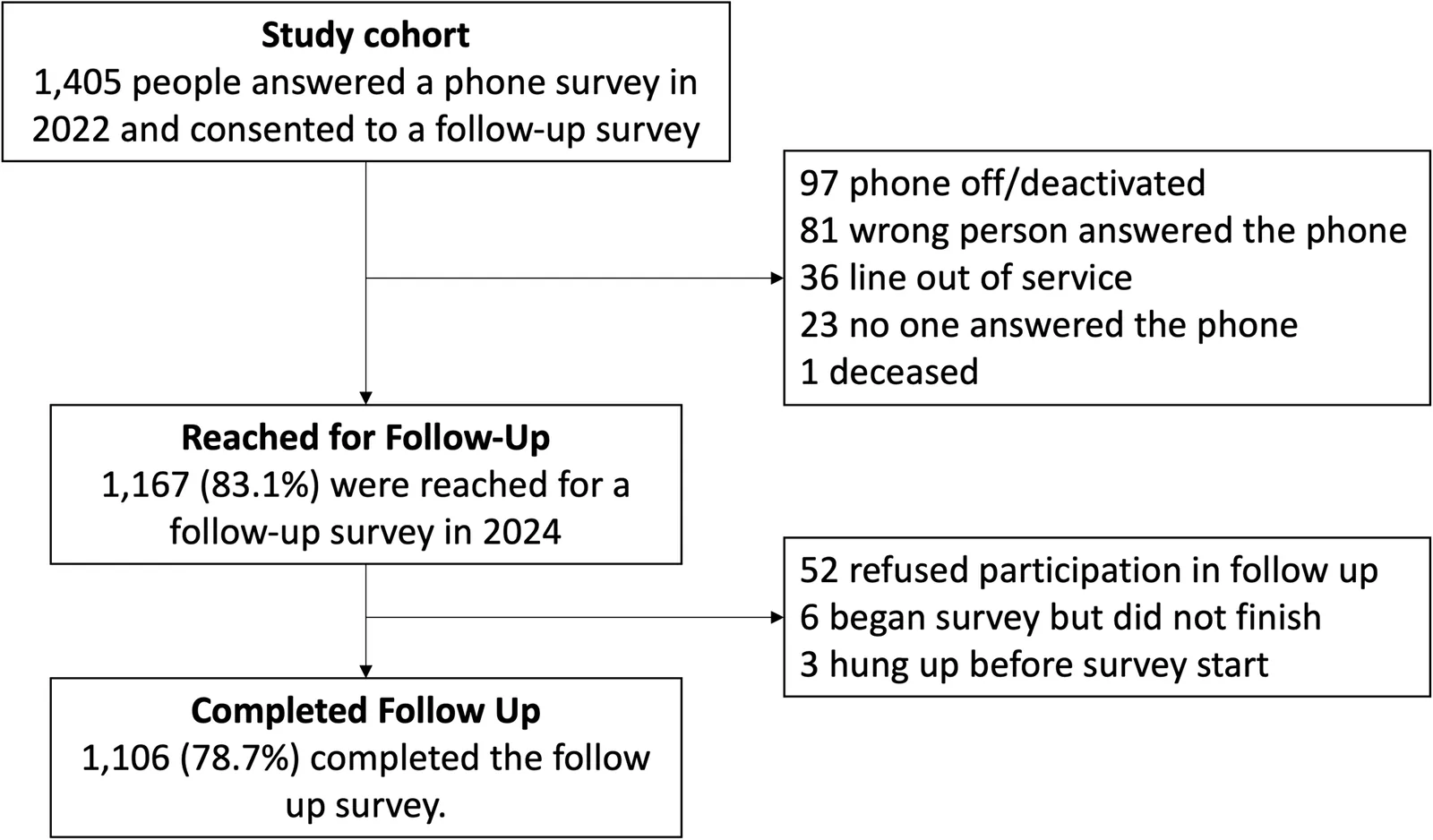
Reasons for non-inclusion in the study outcomes.

In the 2022 survey round, 19688 phones were dialed, 10096 adults were screened for eligibility, 1454 were eligible, 1416 agreed to the 2022 survey, and 1405 consented to follow-up for future surveys. Of these 1405 people, 1167 were successfully re-contacted in 2024 (83.1%), and 1106 were completed the 2024 survey (re-respondents) (78.7%). Of the 238 people we were unable to reach for participation in 2024, the reasons were: no answer or phone off (n = 120), call answered but not by the respondent (n = 81), line out of service (n = 36), or respondent reportedly deceased (n = 1). Of the 61 people we were able to reach in 2024 but were not repeat respondents, the reasons were: declined to participate (52), began the survey but did not finish (9) and hung up before survey began (3). The full breakdown of participant flow between 2022 and 2024 is shown in Fig 1.

Characteristics of the 2022 sample, and their distribution across follow-up outcomes in 2024, are shown in Table 1. Our 2022 sample was comprised of approximately 45.1% men and 54.9% women. Educational attainment varied, with 28.9% having primary education or less, 30.6% having completed secondary school, and 40.5% having pursued education beyond secondary school. Most people were working, but only 10.5% of respondents reported that their household income was sufficient or allowed them to save, while 54.0% of respondents needed to borrow money or use savings in order to meet their expenses. Three-quarters of 2022 respondents were married, 45.2% lived in rural areas while 32.7% lived in towns and 22.1% lived in cities, and 66.3% were non-Catholic Christian.

**Table 1:** Characteristics of the 2022 study sample overall and by 2024 follow-up outcome (Unreached, Retained, and Repeat respondents).

Group	Category	Unretained respondents in 2024	Retained respondents in 2024	Completedfollow-up in 2024	Original 2022 Cohort
	n	238	1167	1106	1405
Age, n (%)	17-32 years	86 (36.1%)	23 (37.7%)	260 (23.5%)	369 (26.3%)
	33-38 years	63 (26.5%)	13 (21.3%)	283 (25.6%)	359 (25.6%)
	39-45 years	62 (26.1%)	12 (19.7%)	298 (26.9%)	372 (26.5%)
	46-84 years	27 (11.3%)	13 (21.3%)	265 (24.0%)	305 (21.7%)
Gender, n (%)	Female	147 (61.8%)	40 (65.6%)	584 (52.8%)	771 (54.9%)
	Male	91 (38.2%)	21 (34.4%)	522 (47.2%)	634 (45.1%)
Residence, n (%)	Urban	57 (23.9%)	16 (26.2%)	237 (21.4%)	310 (22.1%)
	Town	79 (33.2%)	23 (37.7%)	358 (32.4%)	460 (32.7%)
	Rural	102 (42.9%)	22 (36.1%)	511 (46.2%)	635 (45.2%)
Education, n (%)	Primary or less	74 (31.1%)	22 (36.1%)	310 (28.0%)	406 (28.9%)
	Secondary	81 (34.0%)	16 (26.2%)	333 (30.1%)	430 (30.6%)
	Higher	83 (34.9%)	23 (37.7%)	463 (41.9%)	569 (40.5%)
Employment, n (%)	Full-time	45 (18.9%)	12 (19.7%)	285 (25.8%)	342 (24.3%)
	Part-time	8 (3.4%)	2 (3.3%)	39 (3.5%)	49 (3.5%)
	Self-employed/Casual laborer	139 (58.4%)	31 (50.8%)	572 (51.7%)	742 (52.8%)
	Unemployed, Looking	29 (12.2%)	9 (14.8%)	139 (12.6%)	177 (12.6%)
	Unemployed, Not Looking	17 (7.1%)	6 (9.8%)	65 (5.9%)	88 (6.3%)
	Missing	0 (0.0%)	1 (1.6%)	6 (0.5%)	7 (0.5%)
Income, n (%)	Needed Borrow/Savings	115 (48.3%)	28 (45.9%)	615 (55.6%)	758 (54.0%)
	Just Sufficient	93 (39.1%)	25 (41.0%)	369 (33.4%)	487 (34.7%)
	Sufficient/Built Savings	29 (12.2%)	5 (8.2%)	113 (10.2%)	147 (10.5%)
	Missing	1 (0.4%)	3 (4.9%)	9 (0.8%)	13 (0.9%)
Marital status, n (%)	Married/Cohabitating	172 (72.3%)	45 (73.8%)	861 (77.8%)	1078 (76.7%)
	Not-Married/Divorced/. Separated/Widowed	65 (27.3%)	15 (24.6%)	242 (21.9%)	322 (22.9%)
	Missing	1 (0.4%)	1 (1.6%)	3 (0.3%)	5 (0.4%)
Religion, n (%)	Catholic	60 (25.2%)	9 (14.8%)	266 (24.1%)	335 (23.8%)
	Anglican	21 (8.8%)	5 (8.2%)	115 (10.4%)	141 (10.0%)
	Muslim	23 (9.7%)	4 (6.6%)	58 (5.2%)	85 (6.0%)
	Other Christian/Baptist	123 (51.7%)	40 (65.6%)	628 (56.8%)	791 (56.3%)
	African Religion/Other	2 (0.8%)	0 (0.0%)	14 (1.3%)	16 (1.1%)
	None	4 (1.7%)	0 (0.0%)	18 (1.6%)	22 (1.6%)
	Missing	5 (2.1%)	3 (4.9%)	7 (0.6%)	15 (1.1%)

**Fig 2:**
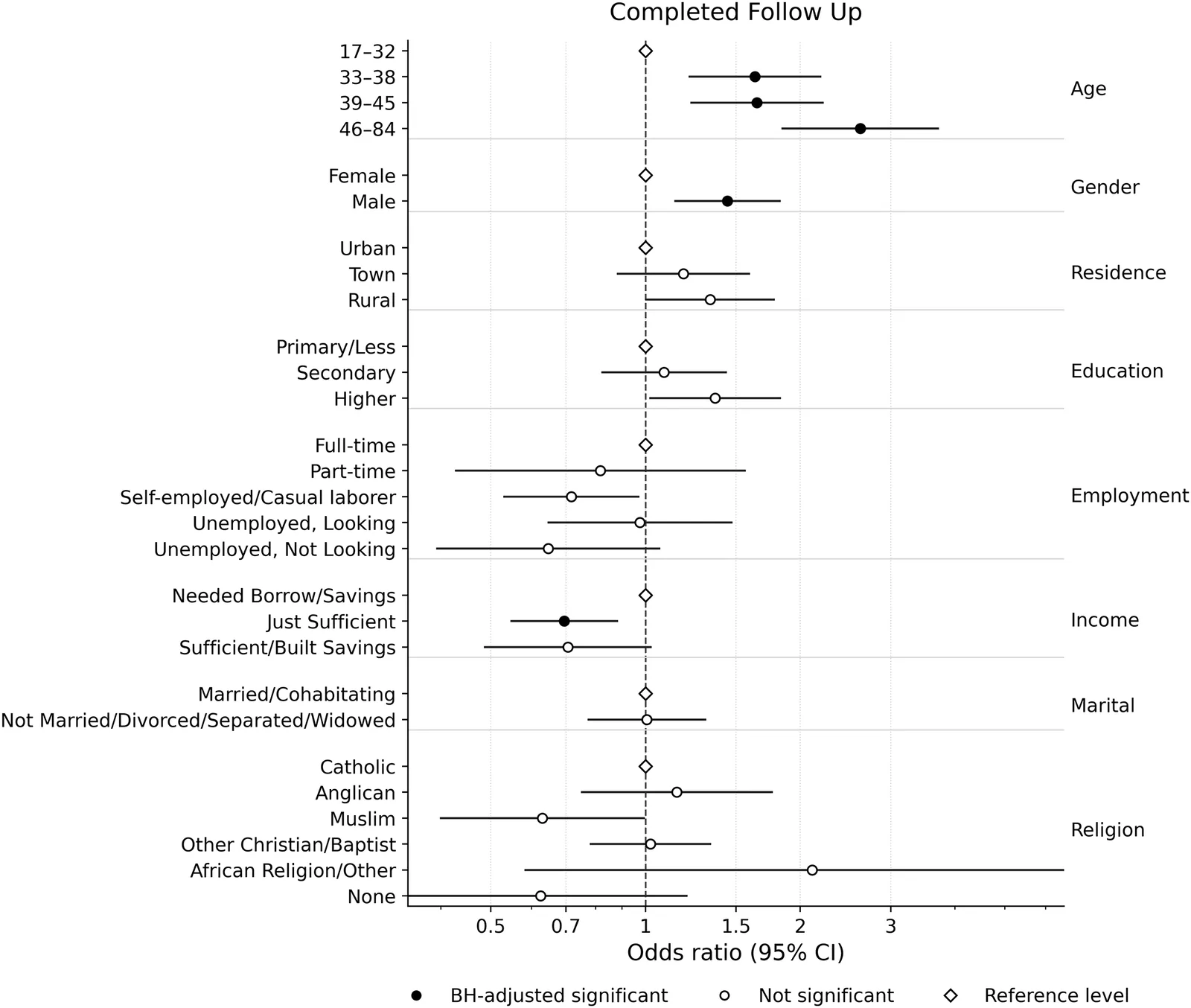
Factors associated with outcomes of completed respondents.

**Fig 3:**
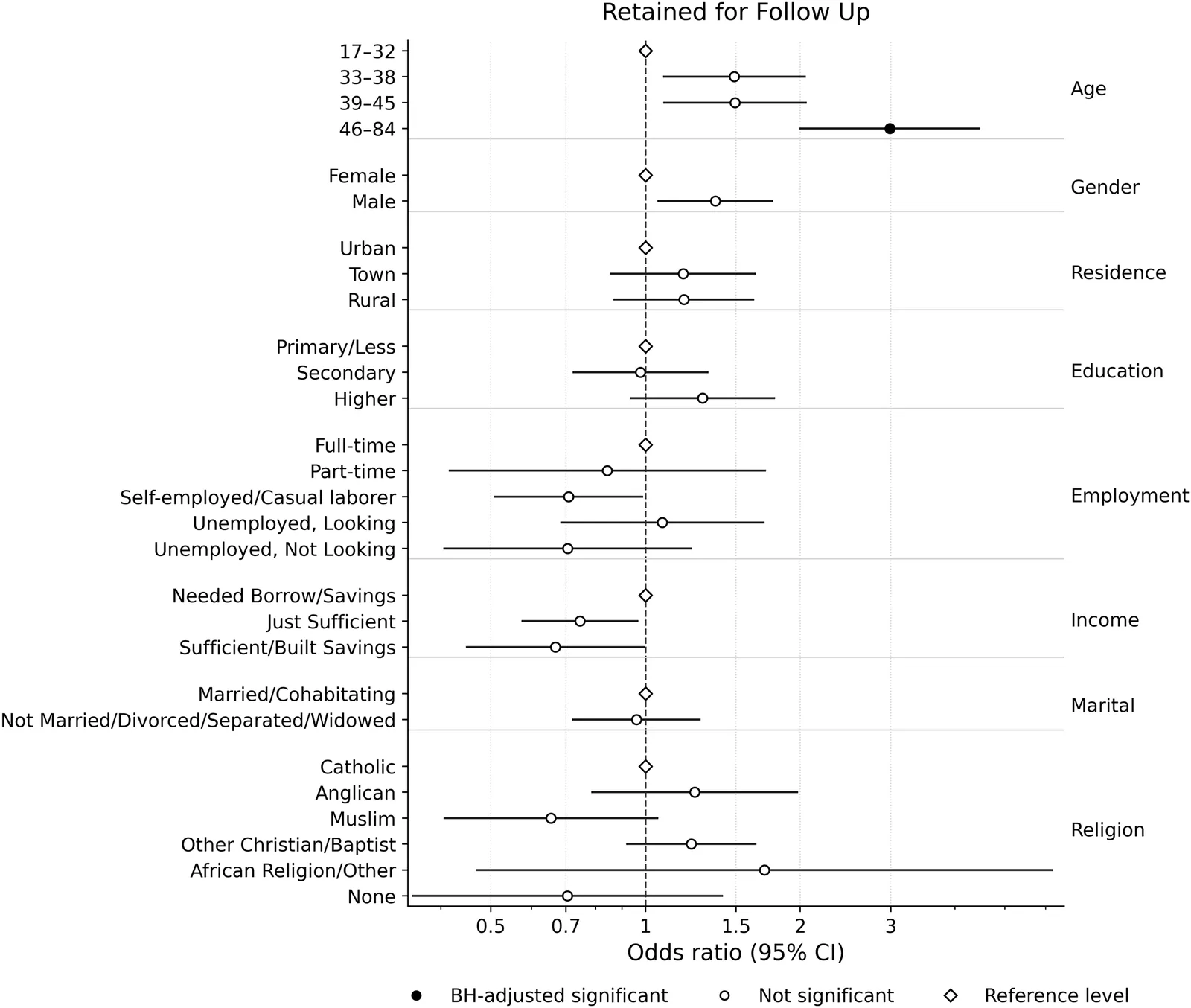
Factors associated with outcomes of retained respondents.

The association between respondent demographics and the completion and retention outcomes are shown in Figs 2 and 3 (see also S2 Table). Participants in all age groups had a higher odds of completing the follow-up survey compared to those aged 17–32 years (33–38 years OR 1.63 [95% CI 1.21–2.20]; 39–45 years OR 1.65 [95% CI 1.22–2.22]; ≥ 46 years OR 2.62 [95% CI 1.84–3.72]) (Fig 2, S2 Table). Men also had higher odds of completing the follow-up survey compared to female respondents (OR 1.44 [95% CI 1.14–1.83]) (Fig 2, S2 Table). People with higher household income sufficiency had lower odds of completing the follow-up survey: compared to those whose recent income was not sufficient, those whose recent income was just sufficient had 0.69 the odds of completing the follow-up survey (95% CI 0.55–0.88) and those whose income was more than sufficient had 0.71 the odds (95% CI 0.48–1.03) (Fig 2, S2 Table). Other characteristics were not significantly associated with this outcome.

Compared with participants aged 17–32 years, respondents aged ≥46 years had significantly higher odds of being retained in the study (OR 2.99 [95% CI 1.99–4.48]) – and no other participant characteristics were associated with the outcome of retention in 2024 (Fig 3, S2 Table).

## Discussion

In this study of Kenyan adults (parents/caregivers of preadolescent girls) which used mobile phone-based data collection, 78.7% of those who participated in a 2022 survey were successfully re-surveyed in 2024. Using a broader definition that also includes those who were re-contacted (but may not have completed the re-survey), 83.1% of people were reached for follow-up. This low attrition rate is very promising for the use of mobile phone-based surveys in LMICs. Longitudinal phone-based surveys from other countries in the region have similarly achieved good retention -- 73% in Lesotho [27], 75% in Tanzania [33], 94.5% in Kenya [37], and 98% in Tanzania [23] – although multi-country surveys and pooled analyses have identified substantial variation across studies [38,39]. Given the high penetration of mobile phones in LMICs, and these numerous studies demonstrating good response rates including for longitudinal surveys, it is likely that studies will increasingly use mobile phone-based data collection. Researchers may wish to deploy techniques to achieve and maintain high survey response rates, including incorporating interactive elements and giving rewards/incentive payments [40].

We found minimal evidence of differential attrition across subgroups, and slight evidence of differential follow-up across subgroups. Only older adults in this sample were significantly more likely to be retained from 2022 to 2024 than their younger counterparts, and no other subgroup had a different pattern of retention. For the main outcome of completing a follow-up survey, we saw a positive association with increasing age and for male respondents versus females, and a negative association with household income. These findings should be considered in light of sample composition; this survey sample was not designed to be representative of the Kenyan general population so may have had unique attributes that affected these results. For example, half of this sample was formally employed in 2022 and over 70% had completed or gone beyond secondary schooling; it is possible that more socioeconomically advantaged individuals are less mobile or less likely to change their phone number, both of which would increase the odds of locating them again two years later. A number of studies from LMICs, including both cross-sectional surveys [12,30,38,41–49] and longitudinal surveys [11,18,27–34], have identified differences in sample composition that may impact the correct interpretation of results from phone-based data collection. However, as mobile phone ownership nears universal levels, the potential for coverage error in mobile phone-based surveys will reduce [41,42]. Recent data from Kenya indicate that 95% of Kenyans own a mobile phone with very few differences across groups [35]. It is nonetheless important to continue to deepen our understanding of different patterns of response to mobile phone surveys and encourage further study on this topic including in-depth qualitative research with both respondents and non-respondents to understand motivations and reasons for patterns of response/non-response.

There are additional limitations to this study that should be noted. We asked respondents only about a few of their sociodemographic characteristics, and it is possible that retention or follow-up response is associated with an unobserved respondent trait. In addition, these respondents are a specific group – Kenyan parents of preadolescent girls – so these results may not generalize to other populations even in the same, or similar, settings. Related, we had limited variation in certain characteristics (like marital status) and this may have limited our ability to detect differences in outcomes for some groups. Lastly, it is possible that participating in the 2022 survey directly affected response rates in 2024; we did not ask participants whether they recalled participating in the earlier survey so cannot assess this relationship.

## Conclusions

In this study with Kenyan adults who were enrolled in 2022 and contacted for re-survey in 2024, 83.1% were reached for follow-up and 78.7% participated in the follow-up survey. This is a very high retention rate that underscores the promise of mobile phone-based data collection, including for longitudinal surveys, in LMICs. We find relatively few differences in patterns of follow-up (only age, gender, and household income); this may be due to the steadily increasing coverage of mobile phones in LMICs because more universal phone ownership may reduce biases in survey outcomes. We encourage more research that uses mobile phones in LMICs, and more research about the use of mobile phone-based surveys in diverse contexts and with diverse populations.

## Supporting information

S1 File Inclusivity in global research. (DOCX)

S2 Table Odds ratios and confidence intervals for all respondent characteristics and both outcomes. (DOCX)

## References

[1] Brick JM. The Future of Survey Sampling. Public Opinion Quarterly. 2011;75(5):872–88. doi: 10.1093/poq/nfr045 PMC4071995 24970951

[2] Hu SS, Balluz L, Battaglia MP, Frankel MR. Improving public health surveillance using a dual-frame survey of landline and cell phone numbers. Am J Epidemiol. 2011;173(6):703–11. doi: 10.1093/aje/kwq442 21343246

[3] AAPOR Cell Phone Task Force. New considerations for survey researchers when planning and conducting RDD telephone surveys in the US with respondents reached via cell phone numbers. Deerfield, IL: American Association for Public Opinion Research. 2010.

[4] Couper MP. The Future of Modes of Data Collection. Public Opinion Quarterly. 2011;75(5):889–908. doi: 10.1093/poq/nfr046

[5] Guterbock TM, Lavrakas PJ, Tompson TN, ZuWallack R. Cost and Productivity Ratios in Dual-Frame RDD Telephone Surveys. Surv Pract. 2011;4(2):1–7. doi: 10.29115/sp-2011-0008

[6] Lynn P, Kaminska O. The Impact of Mobile Phones on Survey Measurement Error. Public Opinion Quarterly. 2012;77(2):586–605. doi: 10.1093/poq/nfs046

[7] Blumberg SJ, Luke JV. Coverage Bias in Traditional Telephone Surveys of Low-Income and Young Adults. Public Opinion Quarterly. 2007;71(5):734–49. doi: 10.1093/poq/nfm047

[8] Brick JM, Dipko S, Presser S, Tucker C, Yuan Y. Nonresponse Bias in a Dual Frame Sample of Cell and Landline Numbers. Public Opinion Quarterly. 2006;70(5):780–93. doi: 10.1093/poq/nfl031

[9] Hensen B, Mackworth-Young CRS, Simwinga M, Abdelmagid N, Banda J, Mavodza C, et al. Remote data collection for public health research in a COVID-19 era: ethical implications, challenges and opportunities. Health Policy Plan. 2021;36(3):360–8. doi: 10.1093/heapol/czaa158 33881138 PMC7928874

[10] Labrique A, Blynn E, Ahmed S, Gibson D, Pariyo G, Hyder AA. Health Surveys Using Mobile Phones in Developing Countries: Automated Active Strata Monitoring and Other Statistical Considerations for Improving Precision and Reducing Biases. J Med Internet Res. 2017;19(5):e121. doi: 10.2196/jmir.7329 28476726 PMC5438457

[11] Zezza A, Martuscelli A, Wollburg P, Gourlay S, Kilic T. Viewpoint: High-frequency phone surveys on COVID-19: Good practices, open questions. Food Policy. 2021;105:102153. doi: 10.1016/j.foodpol.2021.102153 34483442 PMC8405596

[12] Guzman-Tordecilla DN, Vecino-Ortiz AI, Torres-Quintero A, Solorzano-Barrera C, Ali J, Peñaloza-Quintero RE, et al. Examination of the demographic representativeness of a cross-sectional mobile phone survey in collecting health data in Colombia using random digit dialling. BMJ Open. 2023;13(6):e073647. doi: 10.1136/bmjopen-2023-073647 37328185 PMC10277114

[13] Elkasabi M, Khan A. The evolution of mobile phone surveys in low-and middle-income countries: A study of coverage structure. International Journal of Public Opinion Research. 2023;35(4):edad031.

[14] Allen LN, Mackinnon S, Gordon I, Blane D, Marques AP, Gichuhi S, et al. Performance and Resource Requirements of In-Person, Voice Call, and Automated Telephone-Based Socioeconomic Data Collection Modalities for Community-Based Health Programs: A Systematic Review. JAMA Netw Open. 2022;5(11):e2243883. doi: 10.1001/jamanetworkopen.2022.43883 36441550 PMC9706363

[15] International Telecommunication Union. Measuring digital development: Facts and Figures 2024. Geneva, Switzerland: International Telecommunication Union. 2024.

[16] Hoogeveen J, Pape U. Data collection in fragile states: innovations from Africa and beyond. Springer Nature. 2020.

[17] Hersh S, Nair D, Komaragiri PB, Adlakha RK. Patchy signals: capturing women’s voices in mobile phone surveys of rural India. BMJ Glob Health. 2021;6(Suppl 5):e005411. doi: 10.1136/bmjgh-2021-005411 34475116 PMC8413869

[18] Ambel A, McGee K, Tsegay A. Reducing bias in phone survey samples: effectiveness of reweighting techniques using face-to-face surveys as frames. World Bank Group. 2021.

[19] Arita S, Ba MF, Traoré Z, Bonnet E, Faye A, Ridde V. Use of interviewer-administered telephone surveys during infectious disease outbreaks, epidemics and pandemics: a scoping review. BMJ Glob Health. 2023;8(5):e011109. doi: 10.1136/bmjgh-2022-011109 37137536 PMC10163463

[20] Lamanna C, Hachhethu K, Chesterman S, Singhal G, Mwongela B, Ng’endo M, et al. Strengths and limitations of computer assisted telephone interviews (CATI) for nutrition data collection in rural Kenya. PLoS One. 2019;14(1):e0210050. doi: 10.1371/journal.pone.0210050 30699207 PMC6353544

[21] Dang H-AH, Oseni G, Abanokova K. Educational inequalities during COVID-19: Results from longitudinal surveys in Sub-Saharan Africa. International Journal of Educational Development. 2025;112:103174. doi: 10.1016/j.ijedudev.2024.103174

[22] Wollburg P, Markhof Y, Kanyanda S, Zezza A. The evolution of COVID-19 vaccine hesitancy in Sub-Saharan Africa: evidence from panel survey data. BMC Proc. 2023;17(Suppl 7):8. doi: 10.1186/s12919-023-00266-x 37415169 PMC10324117

[23] Dillon B. Using mobile phones to collect panel data in developing countries. J of Intl Development. 2011;24(4):518–27. doi: 10.1002/jid.1771

[24] Nagpal K, Mathur MR, Biswas A, Fraker A. Who do phone surveys miss, and how to reduce exclusion: recommendations from phone surveys in nine Indian states. BMJ Glob Health. 2021;6(Suppl 5):e005610. doi: 10.1136/bmjgh-2021-005610 34380709 PMC8359516

[25] Kempf AM, Remington PL. New challenges for telephone survey research in the twenty-first century. Annu Rev Public Health. 2007;28:113–26. doi: 10.1146/annurev.publhealth.28.021406.144059 17094769

[26] Dabalen A, Etang A, Hoogeveen J, Mushi E, Schipper Y, von Engelhardt J. Mobile phone panel surveys in developing countries: a practical guide for microdata collection. World Bank Publications. 2016.

[27] Lethoko M, Chang C, Badu T, Farley SM, Chen Q, Greenleaf AR. Participation in a 2-year weekly phone surveillance system: results from Lesotho. Surv Pract. 2024;17(1):1–13. doi: 10.29115/sp-2024-000439444694

[28] Ballivian A, Azevedo JP, Durbin W, Rios J, Godoy J, Borisova C. Using mobile phones for high-frequency data collection. Mobile Research Methods. 2015;21.

[29] Demombynes G, Gubbins P, Romeo A. Challenges and opportunities of mobile phone-based data collection: Evidence from South Sudan. 2013.

[30] Banerjee J, Petrosyan S, Rao AR, Jacob S, Khobragade PY, Weerman B, et al. Cohort Profile: Real-Time Insights of COVID-19 in India (RTI COVID-India). BMC Public Health. 2023;23(1):292. doi: 10.1186/s12889-023-15084-1 36759802 PMC9909130

[31] Ratrikaningtyas PD, Lazuardi L, Nugroho A, Wahdi AE, Nurvitasari RI, Azizatunnisa L, et al. Lessons learned from telephone-based data collection for health and demographic surveillance systems during the COVID-19 pandemic in Indonesia. Global Health: Science and Practice. 2024;12(2). 10.9745/GHSP-D-22-00446 PMC11057803 38428996

[32] Himelein K, Kastelic JG. The socio-economic impacts of Ebola in Liberia. 2015.

[33] Hoogeveen J, Croke K, Dabalen A, Demombynes G, Giugale M. Collecting high frequency panel data in Africa using mobile phone interviews. Canadian Journal of Development Studies / Revue canadienne d’études du développement. 2014;35(1):186–207. doi: 10.1080/02255189.2014.876390

[34] Jones L, von Engelhardt J. Insights into the utility and management of mobile phone panel surveys: Evidence from Surveys of Household Resilience in Myanmar. 2020. https://ssrn.com/abstract=3732529

[35] Institute for Development Studies. Summary of results: Afrobarometer Round 10 survey in Kenya. Kenya: University of Nairobi. 2024.

[36] Moucheraud C, Ochieng E, Ogutu V, Chang LC, Golub G, Crespi CM, et al. Intervention-amenable factors associated with lack of HPV vaccination in Kenya: Results from a large national phone survey. Vaccine. 2024;42(26):126410. doi: 10.1016/j.vaccine.2024.126410 39388933

[37] Ashigbie PG, Rockers PC, Laing RO, Cabral HJ, Onyango MA, Mboya J. Phone-based monitoring to evaluate health policy and program implementation in Kenya. Health policy and planning. 2021;36(4):444–53. 33724372 10.1093/heapol/czab029 PMC8128015

[38] Henderson S, Rosenbaum M, Roemer J, Glazerman S, Parkerson D, Warren S. Remote surveying in a pandemic: research synthesis. Innovation for Poverty Action. 2020.

[39] Gibson DG, Pereira A, Farrenkopf BA, Labrique AB, Pariyo GW, Hyder AA. Mobile Phone Surveys for Collecting Population-Level Estimates in Low- and Middle-Income Countries: A Literature Review. J Med Internet Res. 2017;19(5):e139. doi: 10.2196/jmir.7428 28476725 PMC5438460

[40] Fischer F, Kleen S. Possibilities, Problems, and Perspectives of Data Collection by Mobile Apps in Longitudinal Epidemiological Studies: Scoping Review. J Med Internet Res. 2021;23(1):e17691. doi: 10.2196/17691 33480850 PMC7864774

[41] Gibson DG, Wosu AC, Pariyo GW, Ahmed S, Ali J, Labrique AB, et al. Effect of airtime incentives on response and cooperation rates in non-communicable disease interactive voice response surveys: randomised controlled trials in Bangladesh and Uganda. BMJ Glob Health. 2019;4(5):e001604. doi: 10.1136/bmjgh-2019-001604 31565406 PMC6747927

[42] L’Engle K, Sefa E, Adimazoya EA, Yartey E, Lenzi R, Tarpo C, et al. Survey research with a random digit dial national mobile phone sample in Ghana: Methods and sample quality. PLoS One. 2018;13(1):e0190902. doi: 10.1371/journal.pone.0190902 29351349 PMC5774708

[43] Greenleaf AR, Gadiaga A, Choi Y, Guiella G, Turke S, Battle N, et al. Automated and Interviewer-Administered Mobile Phone Surveys in Burkina Faso: Sociodemographic Differences Among Female Mobile Phone Survey Respondents and Nonrespondents. JMIR Mhealth Uhealth. 2020;8(7):e17891. doi: 10.2196/17891 32673250 PMC7388048

[44] Brubaker J, Kilic T, Wollburg P. Representativeness of individual-level data in COVID-19 phone surveys: Findings from Sub-Saharan Africa. PLoS One. 2021;16(11):e0258877. doi: 10.1371/journal.pone.0258877 34788292 PMC8598049

[45] Kibria GMA, Ahmed S, Khan IA, Fernández-Niño JA, Vecino-Ortiz A, Ali J, et al. Developing digital tools for health surveys in low- and middle-income countries: Comparing findings of two mobile phone surveys with a nationally representative in-person survey in Bangladesh. PLOS Glob Public Health. 2023;3(7):e0002053. doi: 10.1371/journal.pgph.0002053 37498841 PMC10374008

[46] Pariyo GW, Greenleaf AR, Gibson DG, Ali J, Selig H, Labrique AB, et al. Does mobile phone survey method matter? Reliability of computer-assisted telephone interviews and interactive voice response non-communicable diseases risk factor surveys in low and middle income countries. PLoS One. 2019;14(4):e0214450. doi: 10.1371/journal.pone.0214450 30969975 PMC6457489

[47] Greenleaf AR, Gadiaga A, Guiella G, Turke S, Battle N, Ahmed S, et al. Comparability of modern contraceptive use estimates between a face-to-face survey and a cellphone survey among women in Burkina Faso. PLoS One. 2020;15(5):e0231819. doi: 10.1371/journal.pone.0231819 32401773 PMC7219703

[48] Pariyo G, Meghani A, Gibson D, Ali J, Labrique A, Khan IA, et al. Effect of the Data Collection Method on Mobile Phone Survey Participation in Bangladesh and Tanzania: Secondary Analyses of a Randomized Crossover Trial. JMIR Form Res. 2023;7:e38774. doi: 10.2196/38774 37079373 PMC10160933

[49] Ndashimye F, Hebie O, Tjaden J. Effectiveness of WhatsApp for Measuring Migration in Follow-Up Phone Surveys. Lessons from a Mode Experiment in Two Low-Income Countries during COVID Contact Restrictions. Social Science Computer Review. 2022;42(2):460–79. doi: 10.1177/08944393221111340

