# Supplementary material for: Understanding retention in longitudinal surveys conducted by mobile phone in lower-resource contexts: A case study from Kenya
Source: PLoS One. 2026 Aug 31;21(8):e0354780. doi: 10.1371/journal.pone.0354780 (PMC13528991; doi:10.1371/journal.pone.0354780)
Supplement: Supplementary file 2 [file pone.0354780.s002.docx]

**S2 Table**: Odds ratios and confidence intervals for all respondent characteristics and both outcomes

| Covariate | Completed follow-up: OR (95% CI) | Retained respondents: OR (95% CI) |
| --- | --- | --- |
| Age: 17–32 | Ref | |
| Age: 33–38 | 1.63 (1.21, 2.20) | 1.49 (1.08, 2.05) |
| Age: 39–45 | 1.65 (1.22, 2.22) | 1.49 (1.08, 2.06) |
| Age: 46–84 | 2.62 (1.84, 3.72) | 2.99 (1.99, 4.48) |
| Employment: Full-time | Ref | |
| Employment: Part-time | 0.82 (0.43, 1.57) | 0.84 (0.41, 1.71) |
| Employment: Self-employed/Casual laborer | 0.72 (0.53, 0.97) | 0.71 (0.51, 0.99) |
| Employment: Unemployed, Looking | 0.98 (0.64, 1.48) | 1.08 (0.68, 1.70) |
| Employment: Unemployed, Not Looking | 0.65 (0.39, 1.07) | 0.71 (0.40, 1.23) |
| Gender: Female | Ref | |
| Gender: Male | 1.44 (1.14, 1.83) | 1.37 (1.06, 1.77) |
| Education: Primary/Less | Ref | |
| Education: Secondary | 1.09 (0.82, 1.44) | 0.98 (0.72, 1.33) |
| Education: Higher | 1.37 (1.02, 1.84) | 1.29 (0.93, 1.79) |
| Income: Needed Borrow/Savings | Ref | |
| Income: Just Sufficient | 0.69 (0.55, 0.88) | 0.75 (0.57, 0.97) |
| Income: Sufficient/Built Savings | 0.71 (0.48, 1.03) | 0.67 (0.45, 1.00) |
| Marital: Married/Cohabitating | Ref | |
| Marital: Not Married/Divorced/Separated/Widowed | 1.01 (0.77, 1.31) | 0.96 (0.72, 1.28) |
| Religion: Catholic | Ref | |
| Religion: Anglican | 1.15 (0.75, 1.77) | 1.25 (0.78, 1.98) |
| Religion: Muslim | 0.63 (0.40, 1.00) | 0.65 (0.40, 1.06) |
| Religion: Other Christian/Baptist | 1.02 (0.78, 1.34) | 1.23 (0.92, 1.64) |
| Religion: African Religion/Other | 2.11 (0.58, 7.65) | 1.70 (0.47, 6.20) |
| Religion: None | 0.63 (0.32, 1.21) | 0.70 (0.35, 1.41) |
| Residence: Urban | Ref | |
| Residence: Town | 1.19 (0.88, 1.60) | 1.18 (0.85, 1.64) |
| Residence: Rural | 1.34 (1.00, 1.79) | 1.19 (0.87, 1.63) |
