# Supplementary material for: Understanding retention in longitudinal surveys conducted by mobile phone in lower-resource contexts: A case study from Kenya
Source: PLoS One. 2026 Aug 31;21(8):e0354780. doi: 10.1371/journal.pone.0354780 (PMC13528991; doi:10.1371/journal.pone.0354780)
Supplement: Supplementary file 3 [file pone.0354780.s004.docx]

We thank the editors and reviewers for their very thoughtful feedback. We have made numerous changes in response to their suggestions, which are detailed below. (All line numbers refer to the clean manuscript file.) We feel the manuscript is much improved as a result and we hope you agree. We look forward to the next steps.

1. In the ethics statement in the Methods, you have specified that verbal consent was obtained. Please provide additional details regarding how this consent was documented and witnessed, and state whether this was approved by the IRB

Thank you, we have added information about this to the manuscript (lines 151-159: “As these were telephone-based surveys, informed consent was obtained orally from all participants. At the beginning of each call, enumerators read a standardized consent script in the respondent’s preferred language, which described the purpose of the study, the voluntary nature of participation, procedures involved, potential risks and benefits, confidentiality protections, and the right to decline or withdraw at any time without penalty. Participants were then asked to indicate their willingness to participate, and oral consent was recorded electronically prior to proceeding with the survey. This consent procedure, including audio recording of consent, was explicitly approved by both Institutional Review Boards.”).

2. Please include a complete copy of PLOS’ questionnaire on inclusivity in global research in your revised manuscript. Our policy for research in this area aims to improve transparency in the reporting of research performed outside of researchers’ own country or community. The policy applies to researchers who have travelled to a different country to conduct research, research with Indigenous populations or their lands, and research on cultural artefacts. The questionnaire can also be requested at the journal’s discretion for any other submissions, even if these conditions are not met. Please find more information on the policy and a link to download a blank copy of the questionnaire here: https://journals.plos.org/plosone/s/best-practices-in-research-reporting. Please upload a completed version of your questionnaire as Supporting Information when you resubmit your manuscript.

We have included this checklist and added mention in the manuscript as recommended (lines 161-163).

3. We note that you have indicated that there are restrictions to data sharing for this study. For studies involving human research participant data or other sensitive data, we encourage authors to share de-identified or anonymized data. However, when data cannot be publicly shared for ethical reasons, we allow authors to make their data sets available upon request. For information on unacceptable data access restrictions, please see <http://journals.plos.org/plosone/s/data-availability#loc-unacceptable-data-access-restrictions>. Before we proceed with your manuscript, please address the following prompts:

If there are ethical or legal restrictions on sharing a de-identified data set, please explain them in detail (e.g., data contain potentially identifying or sensitive patient information, data are owned by a third-party organization, etc.) and who has imposed them (e.g., a Research Ethics Committee or Institutional Review Board, etc.). Please also provide contact information for a data access committee, ethics committee, or other institutional body to which data requests may be sent.

In the online submission form, you indicated that “These data are not publicly available because the research participants did not consent to this, but deidentified data are available from the corresponding author on reasonable request.” All PLOS journals now require all data underlying the findings described in their manuscript to be freely available to other researchers, either 1. In a public repository, 2. Within the manuscript itself, or 3. Uploaded as supplementary information. This policy applies to all data except where public deposition would breach compliance with the protocol approved by your research ethics board. If your data cannot be made publicly available for ethical or legal reasons (e.g., public availability would compromise patient privacy), please explain your reasons on resubmission and your exemption request will be escalated for approval.

The consent forms for this study did not include permission to share data in a repository. We have consulted with both a data librarian and the IRB on this point, and both entities agree that the data cannot be shared; the IRB said “Upon review of the most current protocol and consent forms included in your previously approved application for IRB#22-000005, there is no indication of sharing data, including deidentified data, in a repository.” The IRB that made this determination can be reached at [gcirb@research.ucla.edu](mailto:gcirb@research.ucla.edu).

4. Please include captions for your Supporting Information files at the end of your manuscript, and update any in-text citations to match accordingly. Please see our Supporting Information guidelines for more information: <http://journals.plos.org/plosone/s/supporting-information>.

These are included, thank you.

5. If the reviewer comments include a recommendation to cite specific previously published works, please review and evaluate these publications to determine whether they are relevant and should be cited. There is no requirement to cite these works unless the editor has indicated otherwise.

Not applicable.

Reviewer #1:

6. This is a well-written manuscript on a timely topic that many readers will find useful. While telephone surveys have been extensively studied in Western settings, additional evidence is still needed to document implementation and performance in LMICs. Overall, the paper is clear and easy to follow. The statistical analyses are rigorous, and the findings are broadly consistent with expectations.

Thank you very much for this positive feedback.

7. One suggestion for strengthening the manuscript is to provide more detail on fieldwork and measurement. In particular, I recommend adding: (1) basic information on interview length (e.g., average/median duration and variability), and (2) a brief description of the questionnaire content (key modules or domains), as these factors can meaningfully affect respondent burden and cooperation.

Thank you for this suggestion; we have added this information (lines 106-109: “The surveys asked about knowledge, attitudes and experiences with HPV vaccination and cervical cancer screening for eligible girls and women in the household, as well as demographic and household information. On average the surveys conducted in 2022 were 27.6 minutes in duration (SD 9 minutes).”).

8. In addition, if the 2024 follow-up included any measure of whether respondents remembered the 2022 survey, that information could be incorporated into the analysis. For example, the authors could estimate models separately for respondents who recall the prior survey versus those who do not, or include recall as a covariate or interaction term. This would help clarify whether refusals in 2024 are plausibly related to prior participation experiences in 2022 or driven by other factors.

Unfortunately we did not ask this question in the survey, but it is an interesting idea for future research. We have added mention of this to the limitations section (lines 255-257: “Lastly, it is possible that participating in the 2022 survey directly affected response rates in 2024; we did not ask participants whether they recalled participating in the earlier survey so cannot assess this relationship”).

Reviewer #2:

9. Numerous research activities now leverage on the rapid expansion of cell phone in LMICs to collect data, especially on health-related issues in what is often referred to as m-Health. I share the view of the authors about the fact that its strengths and limitations are still not explored in these settings. This makes this contribution relevant and worth reading. The authors tackle the problem from the angle of cancer prevention and explore retention and loss to follow-up. It is encouraging to read that two years in the parent program, over 80% of participants are still reachable and over 78% still accept to complete the survey. The potential of mobile phone approach on data for time-varying procedures is noted and particularly pertinent. I am familiar with various mobile phone selection approaches, but for the first time, I hear about random-digit dial of mobile phone numbers which makes it closer to a community-based survey: if compatible with ethical considerations, especially regarding privacy, it is worth replicating. There are several questions and modifications to be addressed before the submission could be considered for publication.

Thank you very much for this positive feedback.

10. Abstract: The description of the parent study (cancer prevention) can be more specific (HPV vaccine).

The parent study included questions about both primary and secondary prevention of cervical cancer; we have added the word “cervical” to the Abstract.

11. Abstract: In the results section of this non-structured abstract, there is a very confusing statement on “household income” which the reader will probably not understand.

Thank you for noting this; we have edited the abstract to clarify the outcomes and these edits have hopefully clarified this point (lines 26-30: “Survey re-completion was significantly associated with older age (versus 17-32 year olds: OR 1.63 [95% CI 1.21-2.20] for 33-38 year olds, OR 1.65 [95% CI 1.22-2.22] for 39-45 year olds, and OR 2.62 [95% CI 1.84-3.72] for ≥46 year olds), male gender (OR 1.44 [95% CI 1.14-1.83]), and household income. Only age was significantly associated with our secondary outcome of being retained in the sample for follow-up (regardless of survey re-completion).”).

12. Abstract: The authors seem to have considered retention in the sample as secondary outcome and one would wonder what was the primary outcome.

Thank you for noting this oversight, we have explicitly added this (lines 29-30: “Only age was significantly associated with our secondary outcome of being retained in the sample for follow-up (regardless of survey re-completion)”).

13. Abstract: There is a mention about “subgroups” in the results section which never appears in the methodology and this does not make the abstract a true reflection of the work.

We have clarified that the “subgroups” are the groups aforementioned in the abstract (line 31: “these aforementioned groups”).

14. Introduction: The last paragraph of this section which seems to be about the aim of this report is way too long and could be simplified. Some information there could be moved to the methodology section under study settings, such as the information on the parent study.

We have substantially shortened this section in line with this recommendation (lines 71-76: “In this paper, we seek to describe a study population of Kenyan adults that was selected and enrolled using random-digit dialing of mobile phones; they were first surveyed in 2022 and then followed up with in 2024. We analyze whether there is evidence of differential attrition from the study over this two-year period. Nearly all Kenyans personally own a mobile phone and use it daily; due to this near-universal ownership of mobile phones, Kenya is an optimal environment to understand response and attrition patterns in mobile phone-based surveys.”).

15. Methods: There is no study design.

Thank you for noting this, we have added this information (lines 79: “This is an analysis of data collected from two surveys with a sample of Kenyan adults.”).

16. Methods: This section must contain information on the study settings.

In response to this suggestion, we have added this (lines 87-92: ” Study setting: Kenya is a country of approximately 57 million people in eastern Africa. Per the most recent Afrobarometer data, approximately 95% of Kenyan respondents personally own a mobile phone and 88% of them use it daily; and there are only small subgroup differences: mobile phone ownership was 97.7% among urban respondents and 93.2% among rural respondents, and 95.6% among males and 93.7% among females; this very high coverage of mobile phones made it an appropriate setting for this study about phone survey response rates.”).

17. Methods: The data collection process needs to be elaborated on for the sake of reproducibility: the source of the 25,000 original phone numbers or the process of “random-generation” of phone numbers and “pulling” numbers to call, the process of documenting oral concern (recording?), etc. to me, some of the information in the data collection section of the methods could be moved to the results section.

Thank you for this suggestion to add more information about where the phone numbers originated from; we have added this to the revised manuscript (lines 94-100: “We obtained a list of 25,000 random computer-generated mobile phone numbers, from which we pulled numbers to call. Specifically, we identified valid mobile network prefixes and for each prefix, the remaining digits were populated using a pseudo-random number generator. To avoid systematic bias, numbers were generated independently without replacement, and the final sample was drawn randomly from the pool of successfully reached and eligible respondents. All procedures were pre-specified and implemented programmatically to ensure reproducibility.”). In addition, we have shifted information about sample size/response rate to the Results section (lines 166-175: “In the 2022 survey round, 19688 phones were dialed, 10096 adults were screened for eligibility, 1454 were eligible, 1416 agreed to the 2022 survey, and 1405 consented to follow-up for future surveys. Of these 1405 people, 1167 were successfully re-contacted in 2024 (83.1%), and 1106 were completed the 2024 survey (re-respondents) (78.7%). Of the 238 people we were unable to reach for participation in 2024, the reasons were: no answer or phone off (n=120), call answered but not by the respondent (n=81), line out of service (n=36), or respondent reportedly deceased (n=1). Of the 61 people we were able to reach in 2024 but were not repeat respondents, the reasons were: declined to participate (52), began the survey but didn’t finish (9) and hung up before survey began (3). The full breakdown of participant flow between 2022 and 2024 is shown in Fig 1.”).

18. Methods: Did the authors get phone numbers from local mobile phone operators or something like that?

See our response to comment #18, above, which addresses this point.

19. Methods: In the data analysis section, I don’t understand the need for assigning identifiers to phone numbers which to me are identifiers on their own.

We have clarified that this was to maintain a deidentified dataset (lines 133-135: “We paired respondents’ data from the 2022 and 2024 surveys using a unique identifier assigned to their phone number (a unique identifier was used in order to maintain a deidentified dataset per data protection and privacy restrictions)”).

20. Methods: Was the call in 2024 uniquely for the purpose of this report or was it in terms of timing part of the routine calls planned in the parent survey?

We have added more information about the survey in 2024 (lines 115-117: “This survey replicated questions to the 2022 survey (updated to include girls newly aged-in to HPV vaccination eligibility when relevant) plus a new module about sharing health information within social networks”) which was to collect updated information from respondents about cervical cancer prevention.

21. Methods: The first two paragraphs of the “data analysis” section should actually appear earlier in the methodology.

Thank you for this suggestion; we have restructured the Methods section and added a new subsection on Key Variables (lines 120-131).

22. Results: The first sentence of the results confirms redundance of information as indicated earlier.

We now begin the Results section with information about the study sample so hopefully this is now clearer and not redundant (lines 166-175: “In the 2022 survey round, 19688 phones were dialed, 10096 adults were screened for eligibility, 1454 were eligible, 1416 agreed to the 2022 survey, and 1405 consented to follow-up for future surveys. Of these 1405 people, 1167 were successfully re-contacted in 2024 (83.1%), and 1106 were completed the 2024 survey (re-respondents) (78.7%). Of the 238 people we were unable to reach for participation in 2024, the reasons were: no answer or phone off (n=120), call answered but not by the respondent (n=81), line out of service (n=36), or respondent reportedly deceased (n=1). Of the 61 people we were able to reach in 2024 but were not repeat respondents, the reasons were: declined to participate (52), began the survey but didn’t finish (9) and hung up before survey began (3). The full breakdown of participant flow between 2022 and 2024 is shown in Fig 1.”).

23. Results: Is there any way of describing how “household income” was estimated?

We have added a definition/clarification in the Methods section (lines 129-131: “household income sufficiency over the past 12 months (self-reported insufficient to meet expenses, just enough to meet expenses, or more than enough to meet expenses)”).

24. Results: It sounds like the parent survey has been completed: are the findings available somewhere and referenced in this report?

Thank you for this suggestion, we have added this citation (#36).

25. Results: Else, the results section is well presented. It would have been interesting to better understand the motivations of those participants who cooperated enough to complete the primary survey, but either refused to participate in this survey or hangup their phones: maybe some relations with what the authors refer to as “unobserved respondent trait” in the limitation section.

We agree with this idea, and have added it to the Discussion section as an opportunity for further research (lines 244-247: “It is nonetheless important to continue to deepen our understanding of different patterns of response to mobile phone surveys and encourage further study on this topic including in-depth qualitative research with both respondents and non-respondents to understand motivations and reasons for patterns of response/non-response”).

26. In conclusion, this submission reporting and analyzing a very high retention rate in mobile phone follow-up program would be informative and serve as a guide to the numerous researchers who are conducting or planning to engage into m-Health approaches in health research if properly presented.

Thank you very much.
