# Supplementary material for: Understanding retention in longitudinal surveys conducted by mobile phone in lower-resource contexts: A case study from Kenya
Source: PLoS One. 2026 Aug 31;21(8):e0354780. doi: 10.1371/journal.pone.0354780 (PMC13528991; doi:10.1371/journal.pone.0354780)
Supplement: Peer review history

# Peer review: Decision Letter 0

**DOI:** 10.1371/journal.pone.0354780.r001

**Authors:** Okikiolu Badejo

2 Mar 2026

Dear Dr. Moucheraud,

Thank you for submitting your manuscript to PLOS ONE. After careful consideration, we feel that it has merit but does not fully meet PLOS ONE’s publication criteria as it currently stands. Therefore, we invite you to submit a revised version of the manuscript that addresses the points raised during the review process.

Please submit your revised manuscript by Apr 16 2026 11:59PM. If you will need more time than this to complete your revisions, please reply to this message or contact the journal office at plosone@plos.org. When you're ready to submit your revision, log on to https://www.editorialmanager.com/pone/ and select the 'Submissions Needing Revision' folder to locate your manuscript file.

- A letter that responds to each point raised by the academic editor and reviewer(s). You should upload this letter as a separate file labeled 'Response to Reviewers'.
- A marked-up copy of your manuscript that highlights changes made to the original version. You should upload this as a separate file labeled 'Revised Manuscript with Track Changes'.
- An unmarked version of your revised paper without tracked changes. You should upload this as a separate file labeled 'Manuscript'.

If you would like to make changes to your financial disclosure, please include your updated statement in your cover letter. Guidelines for resubmitting your figure files are available below the reviewer comments at the end of this letter.

If applicable, we recommend that you deposit your laboratory protocols in protocols.io to enhance the reproducibility of your results. Protocols.io assigns your protocol its own identifier (DOI) so that it can be cited independently in the future. For instructions see: https://journals.plos.org/plosone/s/submission-guidelines#loc-laboratory-protocols. Additionally, PLOS ONE offers an option for publishing peer-reviewed Lab Protocol articles, which describe protocols hosted on protocols.io. Read more information on sharing protocols at https://plos.org/protocols?utm_medium=editorial-email&utm_source=authorletters&utm_campaign=protocols.

We look forward to receiving your revised manuscript.

Kind regards,

Okikiolu Badejo, M.D., Ph.D

Academic Editor

PLOS One

Journal Requirements:

When submitting your revision, we need you to address these additional requirements.

1. Please ensure that your manuscript meets PLOS ONE's style requirements, including those for file naming. The PLOS ONE style templates can be found at

https://journals.plos.org/plosone/s/file?id=wjVg/PLOSOne_formatting_sample_main_body.pdf and

https://journals.plos.org/plosone/s/file?id=ba62/PLOSOne_formatting_sample_title_authors_affiliations.pdf

2.  In the ethics statement in the Methods, you have specified that verbal consent was obtained. Please provide additional details regarding how this consent was documented and witnessed, and state whether this was approved by the IRB

3. Please include a complete copy of PLOS’ questionnaire on inclusivity in global research in your revised manuscript. Our policy for research in this area aims to improve transparency in the reporting of research performed outside of researchers’ own country or community. The policy applies to researchers who have travelled to a different country to conduct research, research with Indigenous populations or their lands, and research on cultural artefacts. The questionnaire can also be requested at the journal’s discretion for any other submissions, even if these conditions are not met.  Please find more information on the policy and a link to download a blank copy of the questionnaire here: https://journals.plos.org/plosone/s/best-practices-in-research-reporting. Please upload a completed version of your questionnaire as Supporting Information when you resubmit your manuscript.

4. We note that you have indicated that there are restrictions to data sharing for this study. For studies involving human research participant data or other sensitive data, we encourage authors to share de-identified or anonymized data. However, when data cannot be publicly shared for ethical reasons, we allow authors to make their data sets available upon request. For information on unacceptable data access restrictions, please see http://journals.plos.org/plosone/s/data-availability#loc-unacceptable-data-access-restrictions.

Before we proceed with your manuscript, please address the following prompts:

a) If there are ethical or legal restrictions on sharing a de-identified data set, please explain them in detail (e.g., data contain potentially identifying or sensitive patient information, data are owned by a third-party organization, etc.) and who has imposed them (e.g., a Research Ethics Committee or Institutional Review Board, etc.). Please also provide contact information for a data access committee, ethics committee, or other institutional body to which data requests may be sent.

b) If there are no restrictions, please upload the minimal anonymized data set necessary to replicate your study findings to a stable, public repository and provide us with the relevant URLs, DOIs, or accession numbers. Please see http://www.bmj.com/content/340/bmj.c181.long for guidelines on how to de-identify and prepare clinical data for publication. For a list of recommended repositories, please see https://journals.plos.org/plosone/s/recommended-repositories. You also have the option of uploading the data as Supporting Information files, but we would recommend depositing data directly to a data repository if possible.

Please update your Data Availability statement in the submission form accordingly.

5. In the online submission form, you indicated that “These data are not publicly available because the research participants did not consent to this, but deidentified data are available from the corresponding author on reasonable request.”

All PLOS journals now require all data underlying the findings described in their manuscript to be freely available to other researchers, either 1. In a public repository, 2. Within the manuscript itself, or 3. Uploaded as supplementary information.

This policy applies to all data except where public deposition would breach compliance with the protocol approved by your research ethics board. If your data cannot be made publicly available for ethical or legal reasons (e.g., public availability would compromise patient privacy), please explain your reasons on resubmission and your exemption request will be escalated for approval.

6. Please include captions for your Supporting Information files at the end of your manuscript, and update any in-text citations to match accordingly. Please see our Supporting Information guidelines for more information: http://journals.plos.org/plosone/s/supporting-information.

7. If the reviewer comments include a recommendation to cite specific previously published works, please review and evaluate these publications to determine whether they are relevant and should be cited. There is no requirement to cite these works unless the editor has indicated otherwise.

[Note: HTML markup is below. Please do not edit.]

Reviewers' comments:

Reviewer's Responses to Questions

**Comments to the Author**

1. Is the manuscript technically sound, and do the data support the conclusions?

Reviewer #1: Yes

Reviewer #2: Yes

**********

2. Has the statistical analysis been performed appropriately and rigorously?

Reviewer #1: Yes

Reviewer #2: Yes

**********

3. Have the authors made all data underlying the findings in their manuscript fully available?

The PLOS Data policy

Reviewer #1: Yes

Reviewer #2: Yes

**********

4. Is the manuscript presented in an intelligible fashion and written in standard English?

Reviewer #1: Yes

Reviewer #2: Yes

**********

Reviewer #1: This is a well-written manuscript on a timely topic that many readers will find useful. While telephone surveys have been extensively studied in Western settings, additional evidence is still needed to document implementation and performance in LMICs.

Overall, the paper is clear and easy to follow. The statistical analyses are rigorous, and the findings are broadly consistent with expectations.

One suggestion for strengthening the manuscript is to provide more detail on fieldwork and measurement. In particular, I recommend adding: (1) basic information on interview length (e.g., average/median duration and variability), and (2) a brief description of the questionnaire content (key modules or domains), as these factors can meaningfully affect respondent burden and cooperation.

In addition, if the 2024 follow-up included any measure of whether respondents remembered the 2022 survey, that information could be incorporated into the analysis. For example, the authors could estimate models separately for respondents who recall the prior survey versus those who do not, or include recall as a covariate or interaction term. This would help clarify whether refusals in 2024 are plausibly related to prior participation experiences in 2022 or driven by other factors.

Reviewer #2: Numerous research activities now leverage on the rapid expansion of cell phone in LMICs to collect data, especially on health-related issues in what is often referred to as m-Health. I share the view of the authors about the fact that its strengths and limitations are still not explored in these settings. This makes this contribution relevant and worth reading. The authors tackle the problem from the angle of cancer prevention and explore retention and loss to follow-up. It is encouraging to read that two years in the parent program, over 80% of participants are still reachable and over 78% still accept to complete the survey. The potential of mobile phone approach on data for time-varying procedures is noted and particularly pertinent.

I am familiar with various mobile phone selection approaches, but for the first time, I hear about random-digit dial of mobile phone numbers which makes it closer to a community-based survey: if compatible with ethical considerations, especially regarding privacy, it is worth replicating.

There are several questions and modifications to be addressed before the submission could be considered for publication.

In the abstract:

The description of the parent study (cancer prevention) can be more specific (HPV vaccine).

In the results section of this non-structured abstract, there is a very confusing statement on “household income” which the reader will probably not understand.

The authors seem to have considered retention in the sample as secondary outcome and one would wonder what was the primary outcome.

There is a mention about “subgroups” in the results section which never appears in the methodology and this does not make the abstract a true reflection of the work.

Introduction:

The last paragraph of this section which seems to be about the aim of this report is way too long and could be simplified. Some information there could be moved to the methodology section under study settings, such as the information on the parent study.

In the methodology:

There is no study design.

This section must contain information on the study settings.

The data collection process needs to be elaborated on for the sake of reproducibility: the source of the 25,000 original phone numbers or the process of “random-generation” of phone numbers and “pulling” numbers to call, the process of documenting oral concern (recording?), etc. to me, some of the information in the data collection section of the methods could be moved to the results section.

Did the authors get phone numbers from local mobile phone operators or something like that?

In the data analysis section, I don’t understand the need for assigning identifiers to phone numbers which to me are identifiers on their own.

Was the call in 2024 uniquely for the purpose of this report or was it in terms of timing part of the routine calls planned in the parent survey?

The first two paragraphs of the “data analysis” section should actually appear earlier in the methodology.

Results:

The first sentence of the results confirms redundance of information as indicated earlier.

Is there any way of describing how “household income” was estimated?

It sounds like the parent survey has been completed: are the findings available somewhere and referenced in this report?

Else, the results section is well presented. It would have been interesting to better understand the motivations of those participants who cooperated enough to complete the primary survey, but either refused to participate in this survey or hangup their phones: maybe some relations with what the authors refer to as “unobserved respondent trait” in the limitation section.

In conclusion, this submission reporting and analyzing a very high retention rate in mobile phone follow-up program would be informative and serve as a guide to the numerous researchers who are conducting or planning to engage into m-Health approaches in health research if properly presented.

Great job!

**********

what does this mean?). If published, this will include your full peer review and any attached files.

If you choose “no”, your identity will remain anonymous but your review may still be made public.

**Do you want your identity to be public for this peer review?**  For information about this choice, including consent withdrawal, please see our Privacy Policy

Reviewer #1: **Yes:** Mahmoud Elkasabi

Reviewer #2: No

**********

[NOTE: If reviewer comments were submitted as an attachment file, they will be attached to this email and accessible via the submission site. Please log into your account, locate the manuscript record, and check for the action link "View Attachments". If this link does not appear, there are no attachment files.]

To ensure your figures meet our technical requirements, please review our figure guidelines: https://journals.plos.org/plosone/s/figures

You may also use PLOS’s free figure tool, NAAS, to help you prepare publication quality figures: https://journals.plos.org/plosone/s/figures#loc-tools-for-figure-preparation.

NAAS will assess whether your figures meet our technical requirements by comparing each figure against our figure specifications.

# Author comment: Author response to Decision Letter 1

**DOI:** 10.1371/journal.pone.0354780.r002

2 May 2026

See attached point-by-point response document.

Attachment Submitted filename: RetentionPhoneSurveys_ResponseReviewers.docx

# Editor report: Decision Letter 1

**DOI:** 10.1371/journal.pone.0354780.r003

**Authors:** Laura Kelly

17 Aug 2026

Understanding retention in longitudinal surveys conducted by mobile phone in lower-resource contexts: A case study from Kenya

PONE-D-26-01452R1

Dear Dr. Moucheraud,

We’re pleased to inform you that your manuscript has been judged scientifically suitable for publication and will be formally accepted for publication once it meets all outstanding technical requirements.

Within one week, you’ll receive an e-mail detailing the required amendments. When these have been addressed, you’ll receive a formal acceptance letter and your manuscript will be scheduled for publication.

An invoice will be generated when your article is formally accepted. Please note, if your institution has a publishing partnership with PLOS and your article meets the relevant criteria, all or part of your publication costs will be covered. Please make sure your user information is up-to-date by logging into Editorial Manager at Editorial Manager® and clicking the ‘Update My Information' link at the top of the page. For questions related to billing, please contact billing support.

If your institution or institutions have a press office, please notify them about your upcoming paper to help maximize its impact. If they’ll be preparing press materials, please inform our press team as soon as possible -- no later than 48 hours after receiving the formal acceptance. Your manuscript will remain under strict press embargo until 2 pm Eastern Time on the date of publication. For more information, please contact onepress@plos.org.

Kind regards,

Laura Kelly, PhD

Division Editor

PLOS One

Additional Editor Comments (optional):

Reviewers' comments:

# Editor report: Acceptance letter

**DOI:** 10.1371/journal.pone.0354780.r004

**Authors:** Laura Kelly

PONE-D-26-01452R1

PLOS One

Dear Dr. Moucheraud,

I'm pleased to inform you that your manuscript has been deemed suitable for publication in PLOS One. Congratulations! Your manuscript is now being handed over to our production team.

At this stage, our production department will prepare your paper for publication. This includes ensuring the following:

* All references, tables, and figures are properly cited

* All relevant supporting information is included in the manuscript submission,

* There are no issues that prevent the paper from being properly typeset

You will receive further instructions from the production team, including instructions on how to review your proof when it is ready. Please keep in mind that we are working through a large volume of accepted articles, so please give us a few days to review your paper and let you know the next and final steps.

Lastly, if your institution or institutions have a press office, please let them know about your upcoming paper now to help maximize its impact. If they'll be preparing press materials, please inform our press team within the next 48 hours. Your manuscript will remain under strict press embargo until 2 pm Eastern Time on the date of publication. For more information, please contact onepress@plos.org.

You will receive an invoice from PLOS for your publication fee after your manuscript has reached the completed accept phase. If you receive an email requesting payment before acceptance or for any other service, this may be a phishing scheme. Learn how to identify phishing emails and protect your accounts at https://explore.plos.org/phishing.

If we can help with anything else, please email us at customercare@plos.org.

Thank you for submitting your work to PLOS One and supporting open access.

Kind regards,

PLOS One Editorial Office Staff

on behalf of

Dr. Laura Hannah Kelly

Staff Editor

PLOS One
